# Transcriptome reprogramming of Epstein-Barr virus infected epithelial and B cells reveals distinct host-virus interaction profiles

**DOI:** 10.1038/s41419-022-05327-1

**Published:** 2022-10-22

**Authors:** Nian Ma, Juan Lu, Yonggang Pei, Erle S. Robertson

**Affiliations:** 1 Departments of Otorhinolaryngology-Head and Neck Surgery, and Microbiology, the Tumor Virology Program, Abramson Cancer Center, Perelman School of Medicine at the University of Pennsylvania, Philadelphia, PA USA; 2 Department of Otorhinolaryngology-Head and Neck Surgery, Nanfang Hospital, Southern Medical University, Guangzhou, China; 3 School of Public Health and Emergency Management, Southern University of Science and Technology, Shenzhen, Guangdong China

**Keywords:** Cell biology, Biotechnology

## Abstract

Epstein-Barr virus (EBV) is an opportunistic pathogen that can manifest itself as a potential contributor to human diseases years after primary infection, specifically in lymphoid and epithelial cell malignancies in immune-competent and immune-compromised hosts. The virus shuttles between B cells and epithelial cells during its infection cycle, facilitating its persistence and transmission in humans. While EBV efficiently infects and transforms B-lymphocytes, epithelial cells are not as susceptible to transformation in vitro. We utilized a 3D platform for culturing normal oral keratinocyte cells (NOKs) using Matrigel for greater insights into the molecular interactions between EBV and infected cells. We determined the transcriptome of EBV infected NOKs and peripheral blood mononuclear cells (PBMCs) for 7 and 15 days. LMPs (−1, −2A, and −2B) and EBNAs (−1, −2, −3A, −3B and −3C) were detected in all samples, and lytic gene expression was significantly higher in NOKs than PBMCs. We identified over 2000 cellular genes that were differentially expressed (*P*-value<0.05). Gene ontology (GO) and pathway analyses significantly identified pathways related to collagen-activation, chemokine signaling, immune response, metabolism, and antiviral responses. We also identified significant changes in metalloproteases and genes encoding chemotactic ligands and cell surface molecules. C-X-C chemokine receptor type 4 (CXCR4) was dramatically downregulated in PBMCs and upregulated in NOKs. However, MMP1 was significantly downregulated in NOKs and upregulated in PBMCs. Therefore, multiple pathways contribute to distinct pathologies associated with EBV infection in epithelial and B cells, and MMP1 and CXCR4 are critical molecules involved in regulation of latent and lytic states linked to viral associated diseases.

## Introduction

Epstein-Barr virus (EBV) belongs to the γ-herpesvirus subfamily and establishes a lifelong persistent infection in greater than 95% of adult humans. It is associated with a number of malignancies which include Hodgkin’s lymphoma, Burkitt’s lymphoma, gastric cancers (EBVaGC), and nasopharyngeal carcinoma (NPC) [1–3]. However, the majority of the global population carry EBV as a long-term asymptomatic infection that do not progress to EBV-associated malignancies [4]. Initial infection with EBV occurs in nasopharyngeal epithelial cells to produce progeny, followed by infection of tonsillar B cells to establish lifelong latent infection [5]. The body of knowledge in regards to EBV infection is largely based on in vitro studies, which demonstrated that several latent genes are critical for the transformation of infected B cells, into lymphoblastoid cell lines (LCLs) [6, 7]. Understanding how EBV infection predominantly establishes latent infection in B cells, and lytic replication in epithelial cells will provide important insights into the difference in infection efficacy, as well as the establishment of latent and lytic replication in B and epithelial cells, respectively.

Examination of the biological role of EBV infection in epithelial cells has been hampered due to the lack of an effective infection model. Conventional two-dimensional (2D) monolayer culture is the typical system used to investigate virus-host interactions. However, several aspects of EBV and epithelial cell interactions have been difficult to study in vitro, as the infection efficiency of EBV to epithelial cells is very low in 2D cell culture. Compared to traditional 2D monolayer culture studies, three-dimensional (3D) human cultures offer the possibility of recapitulating the micro-architecture of native tissues and mimicking the biological environment [8]. Cells in vivo are often surrounded by extracellular matrix (ECM), a non-cellular complex network of extracellular molecules, which not only provides a scaffold for the surrounding cells, but also has an important role in cell differentiation, proliferation, survival adhesion, and migration [9]. The Matrigel basement membrane matrix is a commercially available basal cell culture medium containing a gelatinous protein mixture, which is particularly abundant in ECM components and used extensively for 3D cell culture model in vitro. 3D tumor cell cultures provide a useful in vitro experimental platform to study pathogen‐host interactions and can be adapted to examine viral pathogenesis [10, 11]. Recent studies have shown that many viruses, including infectious bronchitis virus (a chicken Coronavirus), avian influenza viruses, herpes simplex virus 1 and human rhinovirus C, could propagate well in this 3D cell culture system using Matrigel as the scaffold [12, 13]. For more precise identification of biologically relevant virus-host interaction information, we need more precise well-characterized datasets. Previously, NOKs was infected with EBV through co-culture with the EBV-positive AKATA BL cell line in vitro [14]. Therefore, in this study we developed a platform for culturing NOKs using a 3D Matrigel culture system to study the interaction between EBV and epithelial cells to obtain more insights into EBV-associated diseases of the epithelium.

In our study, we use RNA-seq to provide unbiased viral and cellular gene transcription profiles from EBV-infected epithelial cells when compared to EBV-infected B cells. We found that EBV infection perturbed the differentiation of these cells, as well as expression of MMP and chemokines families of proteins. We discuss the importance of these findings as they provide new and unique insights in advancing our understanding of the role of EBV and its associated pathogenesis, as well as determination of the fate of infected epithelial and B-cell types.

## Materials and methods

### Ethics statement

Peripheral blood mononuclear cells (PBMCs) from healthy donors were obtained from the University of Pennsylvania Human Immunology Core (HIC). The Core has approval from the Institutional Review Board (IRB) of the University of Pennsylvania and written informed consent was obtained from every donor. All the procedures were conducted according to the declarations of Helsinki protocols [15, 16].

### Cell lines and antibodies

HEK293T (human embryonic kidney cell line) cells harboring BAC GFP-EBV were cultured as previously described [12]. NOKs were obtained from Drs. Rona Scott (LSU, Sherevport, LA) and Jennifer Webster-Cyriaque (UNC, Chapel Hill, NC) [17]. PBMCs were obtained from UPenn human immunology core from de-identified different donors for infection studies and were grown in RPMI 1640 with 10% fetal bovine serum. NPC43 and C17 [18], two latently infected NPC cells (Professor George Tsao, University of Hong Kong), were cultured in RPMI 1640 medium supplemented with 10% fetal bovine serum (Gibco Life Sciences Inc., Grand Island, NY), 4μM of the ROCK inhibitor Y-27632 (Enzo Life Sciences, Farmingdale, NY), 100 U/ml Penicillin and 100 μg/ml streptomycin. Mouse monoclonal antibodies A10 (EBNA3C), S12 (LMP1) and PE2 (EBNA2) were described previously [13, 14].

### Production of BAC GFP-EBV and infection of PBMCs

As previously described [16], HEK293T cells transfected with BAC GFP-EBV were induced to produce virus by culturing for 5 days in complete DMEM medium with Phorbol ester TPA (12-O-tetradecanoylphorbol-13-acetate, 20 ng/ml) and butyric acid (BA, 3 mM); both from Sigma Inc., St. Louis, MO [16]. Viral particles were harvested from induced cultures and concentrated by ultracentrifugation. PBMCs infection was performed as previously described [15].

### Establishment of BAC GFP-EBV infected NOKs 3D cultures

To establish 3D NOKs cultures, Matrigel (BD Biosciences Inc., Baltimore, MD) was poured onto 12-well culture plates to a depth of approximately 0.2 mm, followed by polymerization for 30 min at 37 °C. The mixture of BAC GFP-EBV and single-cell suspension of NOKs were placed on the surface of the Matrigel matrix and incubated at 37 °C overnight. The next day dead cells were removed by aspiration, and a Matrigel layer was overlaid to cover the cells attached to Matrigel at the bottom. After the Matrigel was solidified, the culture medium was added and changed every 2 days. After 2 days the NOKs cells will invade the matrix to form multicellular spheroids. The infection was monitored by GFP expression using a fluorescence microscope. The BAC GFP-EBV infected cells were enriched by selection with puromycin at a concentration of 1 μg/ml.

To recover cells from Matrigel for further culturing, a dispase solution (BD Biosciences, San Jose, CA) was used. After aspirating the culture medium, dispase solution was added to the plate and incubated for 1 h at 37 °C to dissolve Matrigel. The cells were pelleted, washed twice with PBS, resuspended in trypsin/EDTA, and plated back to a 12-well plate for further culturing.

### RNA-sequencing and analysis

Infected PBMCs, NOKs and mock-infected control cells were performed with EBV infection at 0, 7, and 15 days as described above. Total RNA was extracted from EBV-infected PBMCs and NOKs with miRNeasy according to the manufacturer’s protocol (Qiagen Inc., Hilden, Germany). The quality of the extracted RNA was assessed by an Agilent Bio-Analyzer system (Agilent Technologies, Santa Clara, CA). Sequencing libraries were prepared using the TruSeq stranded mRNA kit (Illumina Inc., San Diego, CA) according to the manufacturer’s protocol. RNA-seq data were collected using an Illumina HiSeq3000 platform in single read 50 bp sequencing module at the Genome Sequencing Facility core (Washington University in St. Louis, MO). All samples analyzed passed quality control with the following parameters of the same length (36 bp), 100% coverage in all bases, 25% of A, T, G, and C nucleotide contributions, 50% GC on base content and less than 0.1% over-represented sequences, indicating good quality. Quality check was performed on the raw RNA-Seq reads (FastQC, Site URL), then mapped with the EBV reference genome (the EBV reference genome and annotation were downloaded from https://www.ncbi.nlm.nih.gov/nuccore/ NC_007605.1) and hg38 genome (HISAT2) [19].

### Lentivirus cloning, preparation and infection

The sense strand of MMP1 shRNA is 5′-tcgagtgctgttgacagtgagcgaAGCGGAGAAATAGTGGCCCAGtagtgaagccacagatgtaCTGGGCCACTATTTCTCCGCTgtgcctactgcctcggaa –3′ The sense strand of CXCR4 shRNA is 5′-tcgagtgctgttgacagtgagcgaGGATCAGCATCGATTCCTTCAtagtgaagccacagatgta TGAAGGAATCGATGCTGATCCgtgcctactgcctcggaa–3′.

The upper-case letters designate MMP1 and CXCR4 target sequences, while lower cases specify hairpin and enzyme site sequences. These sense-stranded oligos were annealed with their respective anti-sense stranded oligos and then cloned into the pGIPZ vector with Xho I and Mlu I restriction sites. Besides, a negative control was set using a shControl plasmid including a scrambled control shRNA sequence 5′-TCTCGCTTGGGCGAGAGTAAG–3′ (Dharmacon Research, Chicago, IL). Lentivirus production and transduction have been described previously [20, 21].

### Quantitative RT-PCR

Total RNA was extracted using Trizol reagent (Invitrogen, Carlsbad, CA). The cDNA was generated using Superscript II reverse transcriptase kit (Invitrogen Inc. Carlsbad, CA) according to the manufacturer’s protocol. Quantitative Real-time PCR (qRT-PCR) analysis was performed by using SYBR green Real-time master mix (MJ Research Inc., Waltham, MA). The primers are listed in Supplementary Table 1.

### Western blotting

Whole-cell lysates were prepared by lysing cells in radio-immunoprecipitation assay (RIPA) buffer containing protease inhibitors (Aprotinin, Leupeptin, Pepstatin, and 1 mM phenylmethyl sulfonyl fluoride [PMSF]). They are then probed with appropriate primary antibody, subsequently incubated with corresponding secondary antibody, and visualized on a LiCor Odyssey imager (LiCor Inc., Lincoln, NE). The relative density (RD) of indicated proteins is shown.

### Immunofluorescence

B-cells were airdried and fixed by 4% paraformaldehyde (PFA) including 0.1% Triton X-100 for 15–20 min at room temperature [22]. The fixed cells were washed with 1×PBS three times, and 5% Bovine serum albumin (BSA) was used for blocking. BZLF1 was detected by mouse anti-BZLF1. The slides were examined using an Olympus Fluoview 300 confocal microscope, and images were analyzed by Fluoview software (Olympus Inc., Melville, NY).

### Statistical analysis

The data represented when required are the mean values with standard deviation (SD). The statistical significance was determined for differences in the mean values and was calculated by performing a 2-tailed student’s t-test. *P*-value of <0.05 was considered as statistically significant in all our results (*P < 0.05; **P < 0.01; ***P < 0.001; NS, not significant).

## Results

### EBV infection of PBMCs and NOKs in 3D Matrigel culture system

**Fig. 1:**
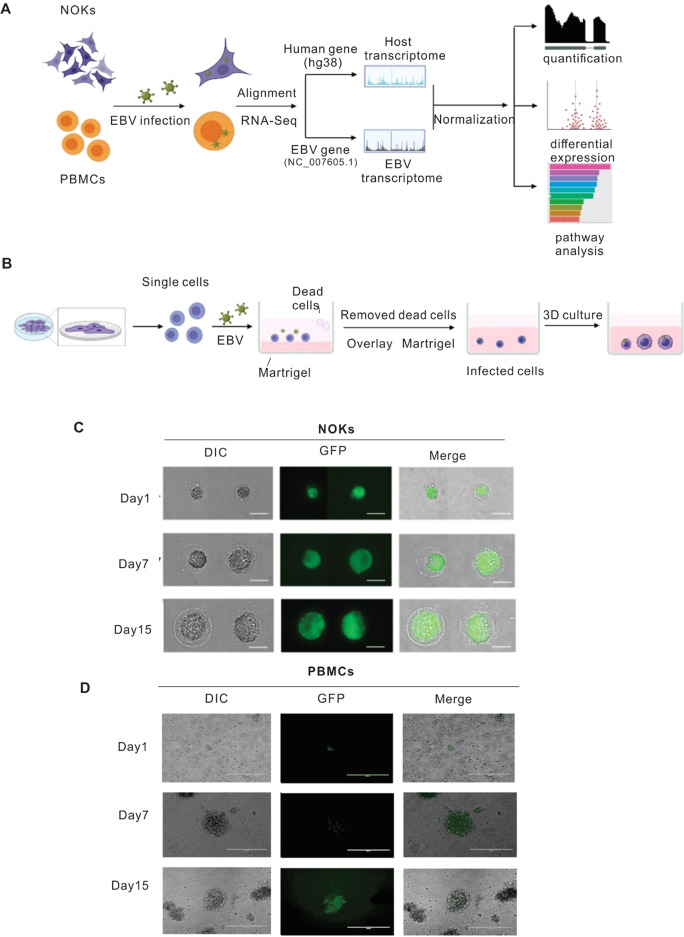
Establishment of EBV-infected NOK-derived 3D culture system and PBMC. Generation and confirmation of EBV infection. To establish 3D NOKs cultures, Matrigel was poured onto 12-well culture plates to a depth of approximately 0.2 mm, followed by polymerization for 30 min at 37 °C. The mixture of BAC GFP-EBV and single-cell suspension of NOKs were placed on the surface of the Matrigel matrix and incubated at 37 °C overnight. The next day dead cells were removed by aspiration, and a Matrigel layer was overlaid to cover the cells attached to Matrigel at the bottom. After the Matrigel solidified, the culture medium was added and changed every 2 days. After 2 days the NOKs cells invade the matrix to form multicellular spheroids. PBMCs were infected with BAC GFP-EBV, then we check the GFP after 7 days and 15 days. **A** Schematic illustration of study design. **B** The flowchart of key procedures to establish BAC GFP-EBV infected NOKs-derived 3D culture system. **C** Representative image of BAC-GFP-EBV infection-mediated GFP expression in NOKs 3D culture at different days (amplification: 400×). **D** Representative image of BAC-GFP-EBV infection-mediated GFP expression in PBMCs culture at different days (amplification: 200×).

EBV primarily infects both epithelial and B cells, with typical production of infectious particles through lytic replication of the virus and establishment of latency in infected B-cells [23, 24]. However, infection of epithelial cells is difficult compared with that of B-cells [25]. The lack of a human epithelial cell model in vitro for studying EBV infection has greatly limited our understanding of the associated epithelial cell pathologies. To enhance the infection efficiency of EBV on epithelial cells, we used a 3D-Matrigel culture system. The work flowchart is shown in Fig. 1A using an ex vivo infection model employing a 3D-Matrigel culture system, as previously described [26]. Once the 3D- Matrigel culture system was successfully generated (Fig. 1B), the EBV-infected NOKs were able to grow within the Matrigel microenvironment and formed spheroids. The cultures were observed daily, the spheroids grew larger in size, and GFP expression (as determined by fluorescence) was observed within the Matrigel matrix suggesting that the NOKs were successfully infected with EBV (Fig. 1C). As expected for PBMCs, it was observed that GFP positive cells appeared enlarged, and formed tight clumps of varying sizes (Fig. 1D) and GFP positivity demonstrated successful infection of PBMCs with EBV.

### EBV gene expression in infected NOKs differs from that seen in infected PBMCs

**Fig. 2:**
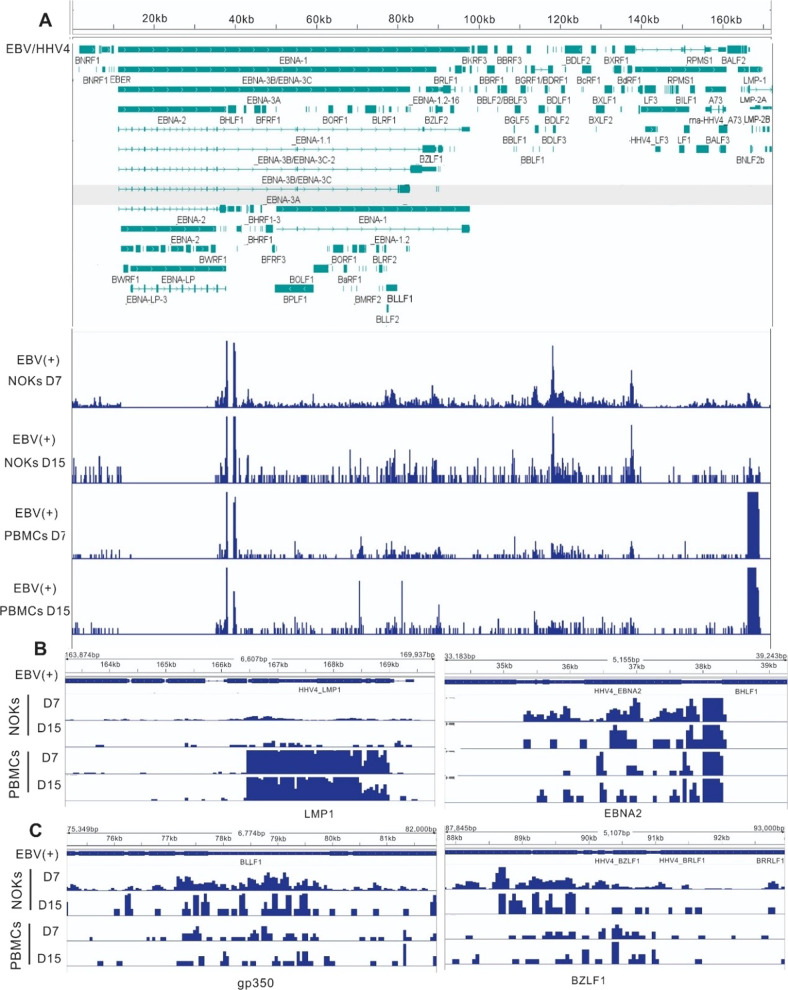
Representative images of sequencing read mapped to EBV-encoded lytic and latent genes in EBV-infected PBMCs and NOKs. EBV RNA expression in NOKs compared to that observed in PBMCs. **A** RNA-seq data for infected NOKs and infected PBMCs at different days mapped to the wild-type EBV genome. Genes corresponding to observed signals are indicated below the tracks. The RNA-seq data image shows that most EBV genes were differentially expressed in NOKs and PBMCs. **B** Zoom in of regions, EBV latent gene LMP1 and EBNA2 from RNA sequencing data shows more detail peaks. **C** Zoom in of regions, EBV lytic gene gp350 and BZLF1 from RNA sequencing data shows more detail peaks.

Viral Gene expression was quantified with reads aligned to the EBV reference genome (NC_007605.1). The EBV transcriptome profiles for each of the samples are shown in Fig. 2A. Varying degrees of latent and lytic gene expression was observed in each sample (with the strongest signals at the loci for EBNA1, EBNA2, BZLF1, BGLF5, and LMP1). The transcripts of 8 selected latent genes were readily detected in all samples, including LMPs and EBNAs (Table 1). The expression levels of the latent transcripts had some significant differences between day 7 and 15 in PBMCs and NOKs after EBV infection. On day 7, the peaks indicating expression levels of EBNA2 and EBNA3C were significantly higher in PBMCs than those in NOKs. LMP2B levels were significantly decreased in PBMCs compared to that seen in NOKs (Fig. 2A and Table 1). Fifteen days after EBV infection, the expression levels of LMP1 and EBNA2 were higher in PBMCs than those in NOKs, and LMP1 levels in PBMCs were reduced significantly on day 15 post-infection (Fig. 2B). In contrast to latent genes, the expression levels of most of the lytic genes were higher in NOKs than those in PBMCs after EBV infection with the highest levels of BZLF1 and gp350 post-infection (Fig. 2C and Table 1B). In NOKs, some of the lytic genes were upregulated at 7 days and increased in levels at day 15 post-infection. These include BALF4, BALF5, BCRF1, and BILF1. However, the expression levels of BBRF3, BGLF3 and BLRF1 were upregulated on day 7 and then repressed on day 15 (Table 1C). In PBMCs, there was no significant difference for lytic gene expression between days 7 and 15 post-infection. Compared with PBMCs, the expression of several lytic genes was significantly increased in NOKs on day 15, including BALF5, BCRF1, BHRF1, BILF1 and BMRF1 (Table 1C). To see the expression profiles of the other differentially expressed EBV genes in these two cell lines, see Supplementary Tables 2 and 3. These results strongly suggest that EBV infection mainly establishes lytic replication in epithelial cells and latent infection in B cells.

**Table 1:** The expression of selected EBV-encoded genes in both NOKs and PBMCs after EBV infection.

	Mean expression level (CPM)			
	EBV-infected NOKs		EBV-infected PBMCs	
A. latent genes				
Gene name	Day 7	Day 15	Day 7	Day 15
LMP-1	32600.23	42192.05	291272.10	276162.89
LMP-2A	3211.37	1939.86	1535.71	3488.37
LMP-2B	4379.14	3394.76	511.90	581.39
EBNA-1	19657.45	21338.51	7934.48	11627.92
EBNA-2	22382.25	24733.27	53493.74	53488.37
EBNA-3A	17516.54	12124.15	22011.77	14534.88
EBNA-3B	26177.50	29582.93	24827.24	19767.44
EBNA-3C	19462.82	15033.95	31481.96	26162.79

B. lytic genes				
Gene name	Day 7	Day 15	Day 7	Day 15
BZLF1	10120.67	11639.19	7166.62	4069.76
BALF4 (gp110)	15083.69	10184.28	5119.02	1860.65
BRLF1	9050.21	5819.59	4095.21	3488.37
BLLF1 (gp350)	36590.11	40737.15	16124.90	21511.63

C. Compared the expression of EBV genes in NOKs and PBMCs								
	EBV (+) NOKs D15 vs D7		EBV (+) PBMCs D15 vs D7		NOKs vs PBMCs at D7		NOKs vs PBMCs at D15	
Gene name	fold change	*P* value	fold change	*P* value	fold change	*P* value	fold change	*P* value
BALF4	1.67	0.02	ND	ND	ND	ND	ND	ND
BALF5	2.28	0.01	ND	ND	ND	ND	1.99	0.01
BBRF3	−2.44	0.01	ND	ND	ND	ND	ND	ND
BCRF1	3.48	0.01	ND	ND	ND	ND	3.37	0.01
BHRF1	ND	ND	ND	ND	3.69	5.66E-07	2.88	0.01
BILF1	1.60	0.03	ND	ND	ND	ND	3.68	0.01
BGLF3	−6.59	0.01	ND	ND	ND	ND	ND	ND
BLRF1	−3.07	0.03	ND	ND	ND	ND	−3.59	0.01
BMRF1	ND	ND	ND	ND	ND	ND	1.76	0.03
BZLF1	ND	ND	ND	ND	2.20	0.02	ND	ND

*ND* no difference.

### RNA sequencing reveals that EBV infected NOKs have distinctly different host transcriptional landscapes compared to infected PBMCs

**Fig. 3:**
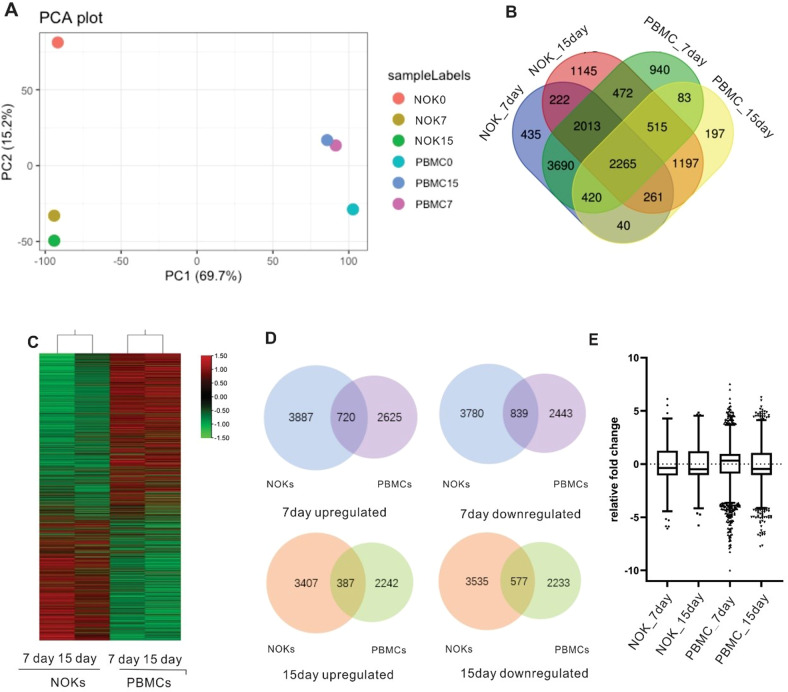
EBV infection leads to distinctly different patterns of gene expression in NOKs and PBMCs. Principal Component Analysis based on RNA-Seq analysis of NOKs and PBMCs. Host genes are differentially expressed between NOKs and PBMCs. **A** Relationship between the samples based on cellular gene expression using the Principal Component Analysis. The gene expression values are normalized to the whole dataset. **B** Cross analysis Venn diagrams of the results using combination of the infected NOKs and PBMCs. Venn diagram showing the total number of gene expressed in different samples. The number of genes differentially expressed in NOKs and PBMCs. The Venn diagram was created using draw Venn diagram. **C** Hierarchical clustering (shown as a heatmap) of differentially expressed genes between NOK infected cells and PBMC infected cells. Red and green colours represent relative high and low log2 gene expression values, respectively. **D** Venn diagrams comparing the numbers of genes observed to be up-regulated or down-regulated by EBV infection in NOKs (adjusted *P* < 0.01) compared with PBMCs (adjusted *P* < 0.01) at day7 and day15. **E** Identify infected NOKs and PBMCs with different temporal profiles. The box plot shows the fold change in expression of the main genes per time point.

We mapped RNA-Seq reads to the Genome Reference Consortium human genome (GRCh38), correlations between samples were viewed by Principle Component Analysis (PCA), and the PCA plots showed that NOKs, PBMCs, and control were clustered in diverse groups which showed an effect of EBV infection (Fig. 3A). Overall, when reads that were shared between replicates were mapped for *P*-value <0.05 in gene expression, thousands of genes, were up or downregulated in different groups. To investigate the different host cell changes induced by EBV infection in both cell lines, we determined the overlap of these four sets with the changed genes. Our results indicated that 2265 genes with significant changes (*P* < 0.05) had simultaneously altered expression levels in these four groups (Fig. 3B). 2265 genes showed an opposite pattern of expression in NOKs and PBMCs, suggesting that vastly different transcriptional changes occurred following EBV infection in the two cell types (Fig. 3C). Because these observed changes are large in magnitude and many in number in different groups, we observed the overlap of numerous genes in NOKs and PBMCs after EBV infection (Fig. 3D). The overlap was more extensive for 7days (720 and 839), whereas only 387 and 577 gene changes were common between the two group data sets at 15days. Interestingly, comparing all NOKs and PBMCs genes measured, their expression was more strongly induced in NOKs than in PBMCs (Fig. 3D). As seen from the boxplot representation, the gene expression in PBMCs displayed greater fold changes than that seen in NOKs (Fig. 3E). Although EBV had a greater effect on gene expression in infected NOKs, differences were much greater in infected PBMCs. EBV infection of these two different cell lines may induce different biological processes in the cells, which can impact their regulatory function. These results demonstrate that EBV has broader effects on gene transcription in NOKs. However, EBV infection drives changes to a range of gene expression profiles that affects specific biological processes in PBMCs.

### EBV-infection of PBMCs and NOKs induces changes in transcription profiles of genes related to major biological processes

**Fig. 4:**
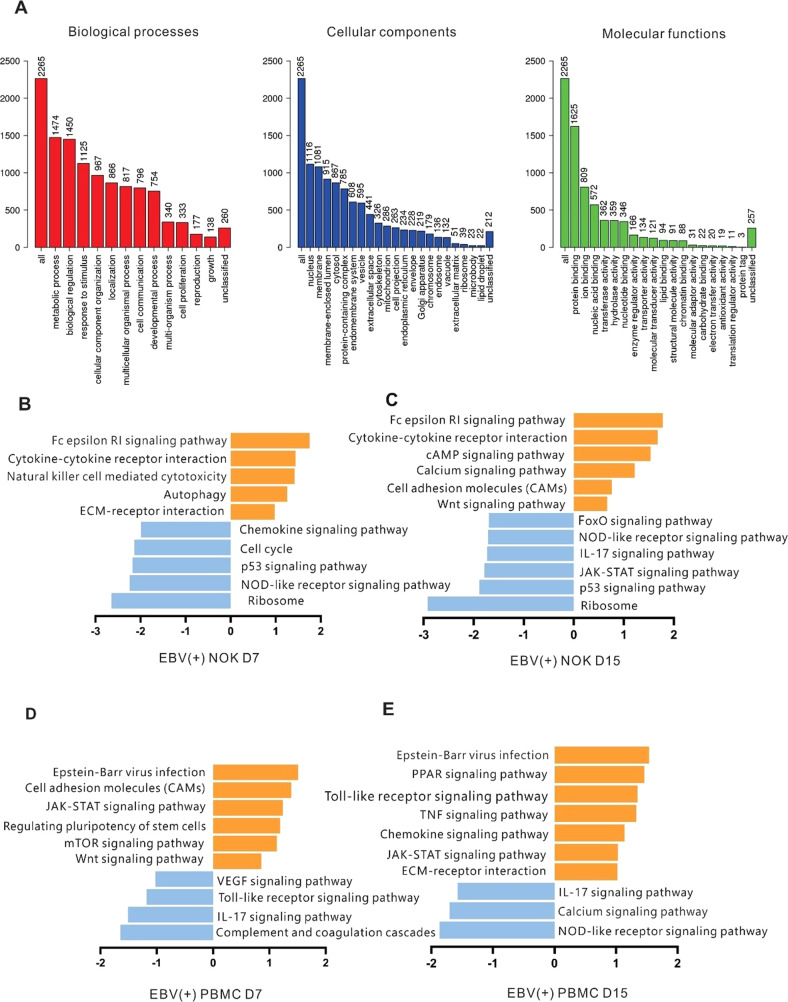
Gene ontology (GO) and pathway analyses. Canonical pathways associated with human gene expression changes by RNA-Seq in infected NOKs and infected PBMCs at 7days and 15days when compared to the mock infected cells. **A** Gene ontology (GO) analyses. Bar charts are shown for GO Slim analysis terms for differentially transcribed protein-coding genes showing a change in both infected NOKs and infected PBMCs compared to no infected cells. **B**–**E** Gene set enrichment analysis (GSEA) for KEGG pathways identified from differentially transcribed protein-coding genes after BAC GFP-EBV infection. KEGG pathways are plotted against their average normalized enrichment score, showing upregulated (orange) and downregulated (blue) pathways. The width of the bars reflects the *p*-value, and the boxes reflect the ratio of the number of genes in the data set represented in the pathway.

We performed a gene enrichment analysis to understand changes in the biological processes due to global transcriptional changes post-EBV infection of the host cells. Specifically, the 2265 identified genes were linked to numerous biological processes, including those involved in metabolism, cell communication, and cell proliferation. The molecular functions of the gene products, those correlated with protein/ion/nucleic-acid/nucleotide/lipid/chromatin binding and transferase/hydrolase/enzyme regulator functions were all upregulated (Fig. 4A). Analysis of the KEGG database using overrepresentation analysis (ORA-KEGG), showed involvement of the p53 signaling pathway, cell cycle, apoptosis, Fox0 signaling pathway, Epstein-Barr virus infection, and pathways involved in cancer (Table 2). Differentially expressed genes with a 2-fold or greater expression change were sorted into categories based on the canonical pathways using GSEA of the KEGG database (GSEA-KEGG). The analyzed transcripts were differentially regulated at the two time points of infected NOKs. They include transcripts for natural killer cell mediated cytotoxicity and cytokine-cytokine receptor interaction (related primarily to innate immune responses) that were upregulated by 7 days, followed by upregulation of the WNT signaling pathway, the cAMP signaling pathway and calcium signaling pathway involved in cancer development at 15 days post-infection (Fig. 4B, C). To further investigate how EBV modulates B-cell responses after infection, we analyzed the potential pathways underlying the differential gene expression. Host genes involved in the cell adhesion molecules, JAK-STAT signaling pathway and chemokine signaling pathway were significantly regulated following infection (Fig. 4D, E). Notably, the chemokine signaling pathway, the JAK-STAT signaling pathway and the calcium signaling pathway affected by EBV infection presented opposite tendencies in NOKs and PBMCs. This likely accounts for the ability of EBV to drive NOKs and PBMCs towards different cell fates.

**Table 2:** ORA-KEGG pathways transcriptionally regulated in NOKs and PBMCs.

Pathway	*P* value	FDR
p53 signaling pathway	5.13E-06	4.18E-04
Cell cycle	1.21E-07	3.18E-05
Apoptosis	1.95E-07	3.18E-05
Autophagy	2.57E-06	2.79E-04
Fox0 signaling pathway	3.76E-05	1.75E-03
Epstein-Barr virus infection	4.68E-05	1.91E-03
Pathways in cancer	3.05E-05	1.66E-03

Transcriptionally regulated protein-coding genes induction by EBV infection in NOKs and PBMCs were subjected to overrepresentation analysis (ORA). FDR, false-discovery rate.

### Expression of MMP protein family members vacillate between epithelial and B-cells with MMP1 important for latent infection of epithelial cells

**Fig. 5:**
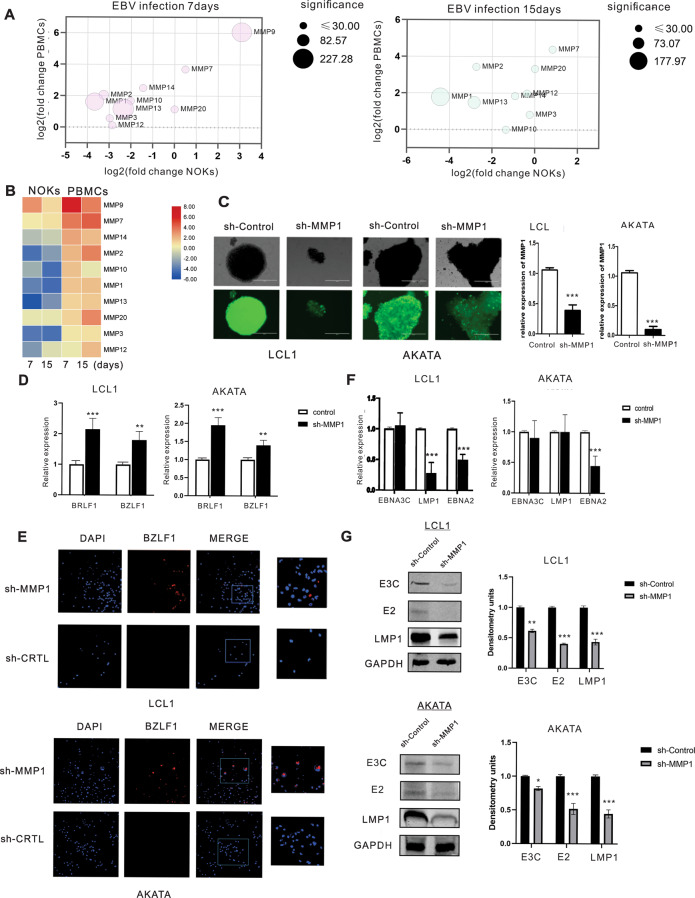
The expression levels of MMP family showed an opposite trend in the two infection models and MMP1 is important for maintenance of the latent infection. Expression of the MMP protein family vacillate between the two infection systems and MMP1 is important for maintaining latency in epithelial cells. **A** Bubble plot that displays genes of MMP protein family. Axis values represent log 2-transformed fold change of transcript expression in PBMCs compared to NOKs. The size of the bubble corresponds to the log 10-transformed adjusted p-value to denote the statistical significance of differential expression. The figure shows that most of MMP protein family shows two distinct expression patterns after EBV infected NOKs and PBMCs. **B** Heat map of differentially expressed MMPs in NOK infected cells and PBMC infected cells. Heat map shows that most of MMPs exhibited a decreasing expression trend in infected NOKs, but they were up-regulated in infected PBMCs. **C** MMP1 knocked down LCL1 and AKATA cells were constructed by lentiviruses and selected by puromycin for three weeks. GFP fluorescence was determined in the selected cells. Validation of MMP1 expression by real time-PCR. **D** Quantification of lytic gene BZLF1 and BRLF1 mRNA from three experiments is shown. **E** Immunofluorescence staining was performed to detect BZLF1 expression in LCL1 and AKATA cells. **F** Quantification of latent gene EBNA3C, LMP1 and EBNA2 mRNA from three experiments is shown. **G** The expression of EBV latent proteins was detected in MMP1-knockdown LCL1 and AKATA cell lines with WB. The relative density (RD) of protein was quantitated and shown.

Matrix metalloproteinases (MMPs) participate in tissue repair after acute injury, as well as participate in the development of cancer by promoting a pro-tumorigenic microenvironment [27]. Studies have also reported that MMPs can play critical roles in progression of cancers [28]. The MMP family of transcripts rises sharply when PBMCs are infected with EBV (Fig. 5A). In contrast, it showed a persistent decline in NOKs. Therefore, we wondered whether they may have a role in regulating latent infection. We focused on MMP1 as it was ranked top among all the down-regulated genes in infected epithelial cells (Table 3). To provide more details, among most statistically significant differences observed in our dataset were the downregulation of MMP1, MMP2, and MMP13 in NOKs after EBV infection 7days. Meanwhile, the expression of these genes remained reduced at 15days in NOKs, but they showed a continuous increase in expression levels in infected PBMCs (Fig. 5A, B). To further investigate, we knocked down MMP1 in LCL1 and AKATA cells to determine if it has any effect on viral reactivation as seen by lytic gene expression. Lentiviruses encoding shMMP1 were used to transduce LCL1 and AKATA cells. The transduction by shMMP1 encoding lentiviruses was confirmed by visualizing green fluorescence protein. Knockdown of MMP1 transcripts was analyzed by real-time PCR using gene-specific primers (Fig. 5C). A significantly higher level of the immediate early genes BZLF1 and BRLF1 expression was observed in shMMP1 LCL1 cell lines compared to the shControl cells. A similar effect was also observed in the shMMP1 AKATA cell group set (Fig. 5D). It was further validated through immunofluorescence analysis which showed that BZLF1 expression was upregulated in shMMP1 LCL1 and AKATA cell lines (Fig. 5E). We further examined whether MMP1 was involved in regulating EBV latency in EBV positive cell lines. Our results showed that knockdown of MMP1 led to a decrease of the EBV-encoded latent gene transcript LMP1 and EBNA2 in LCL1, and EBNA2 predominantly in AKATA cells (Fig. 5F). Furthermore, a significant drop in the protein levels of EBNA2, EBNA3C and LMP1 were observed (Fig. 5G). Therefore, MMP1 may have a role in the maintenance of EBV latent infection and in development of associated cancers.

**Table 3:** Most significant differentially transcribed RNAs in NOKs after 7 and 15 EBV infection.

	NOKs		NOKs	
	EBV (+) D7 VS EBV (−)		EBV (+) D15 VS EBV (−)	
Gene Name	Fold change	*P*-value	Fold change	*P*-value
**Upregulated**				
LCN2	3.77	8.53E-113	4.39	1.28E-149
SLC6A14	3.31	1.37E-20	4.34	8.13E-30
SMOC1	3.66	5.15E-50	3.51	5.26E-39
DAPK1	3.41	2.73E-45	3.75	1.37E-47
STEAP4	4.06	1.3E-67	4.61	1.15E-99
GOLGA8J	3.40	1.41E-32	3.66	9.67E-31
SLAMF7	3.34	1.49E-23	3.66	1.64E-21
MUC4	4.78	8.18E-210	4.83	2.01E-185
MUC16	6.12	2.59E-22	4.54	3.4E-13
VNN1	5.52	1.42E-69	3.95	3.15E-22
**Downregulated**				
DIAPH2	−3.29	6.07E-18	−2.74	5.18E-11
SLITRK6	−3.14	1.45E-25	−3.24	2.85E-22
MMP1	−3.47	1.84E-175	−4.15	1.08E-178
S100A2	−3.67	7.22E-226	−3.80	2.65E-194
KRT6A	−6.05	4.68E-112	−5.76	3.55E-87
GPX2	−4.29	1.01E-64	−3.47	5.53E-42
KCNH3	−3.21	7.36E-13	−3.02	3.19E-11
PTHLH	−4.00	3.23E-197	−3.89	1.91E-144
KRT14	−5.28	2.53E-190	−4.73	6.03E-131
EPGN	−4.24	9.06E-66	−3.62	2.61E-45

### MMP1 restricts EBV lytic reactivation and provides growth-supportive properties in infected epithelial cell lines

**Fig. 6:**
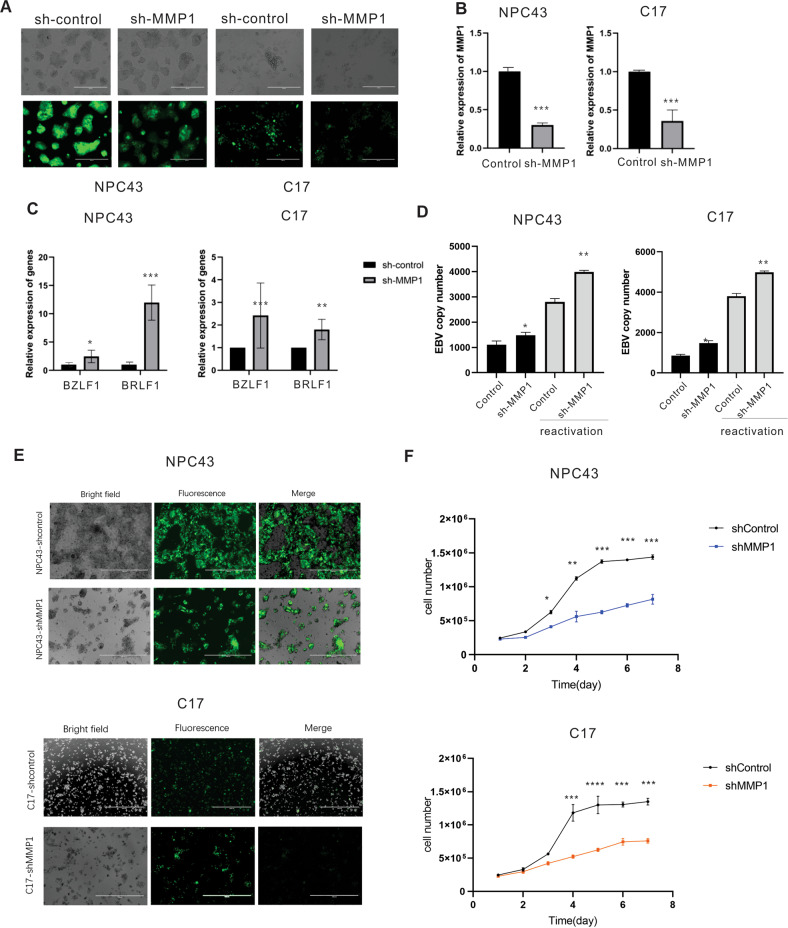
MMP1 is important for maintenance of latent infection in epithelial cells. MMP1 restricts EBV lytic reactivation and provides growth-supportive properties in infected epithelial cell lines. **A** MMP1 knocked down NPC43 and C17 cells were constructed by lentiviruses and selected by puromycin for three weeks. GFP fluorescence was determined in the selected cells. **B** The expression of endogenous MMP1 was determined by qRT-PCR. **C** Quantification of EBV lytic gene BZLF1 and BRLF1 mRNA from three experiments is shown. **D** Relative EBV-GFP DNA copy numbers were quantified as a measure of EBV load released from NPC43-shMMP1 and C17-shMMP1 before and after the reactivation with TPA and Butyrate. EBV DNA copy number was detected by real-time qPCR. **E** Cell proliferation fluorescence microscopy images of NPC43-shControl, NPC43-shMMP1, C17-shControl and C17-shMMP1 after 5 days. **F** Cell growth curves of NPC43 and C17 cells determined by cell count after MMP1 knockdown at different days.

To further characterize the phenotype of epithelial cells after EBV infection suggested by our transcriptomic data, we performed follow up in vitro experiments to assess the functional consequences of MMP1 on recently derived, latent NPC cells. The stable MMP1 knocked down NPC43 and C17 cells were generated with lentivirus transduction and puromycin selection as described above (Fig. 6A). The relative expression of MMP1 was confirmed by RT-PCR showing knockdown of MMP1 transcripts (Fig. 6B). Our result showed that the immediate early lytic genes BRLF1 and BZLF1 were increased in shMMP1 knockdown in the two NPC cells NPC43 and C17 compared with shControl cell lines (Fig. 6C). To determine whether shRNA-mediated silencing of MMP1 resulted in induction of complete EBV lytic replication, the level of EBV-DNA released into culture supernatants was quantified in C17 and NPC43 cells. The control NPC43 and C17 cells produced relatively low levels of spontaneous EBV genome copies in the supernatant, whereas a consistent increase in the levels of EBV-DNA was detected in the supernatant of the cell cultures 72 h after reactivation. The differences between the shControl cells and shMMP1 cells, for NPC43 and C17 were similar without and with reactivation, with the levels of virions collected in the supernatant being significantly higher in the MMP1 knockdown group compared to the control group (Fig. 6D). MMPs affect the biochemistry, migration, proliferation and survival of cells, and inhibiting MMP activity promotes scar formation [29, 30]. Therefore, we wanted to further determine whether MMP1 would affect the cell growth of NPC cells. Our results showed that knockdown of MMP1 notably suppressed cell growth (Fig. 6E, F). These results suggested MMP1 significantly decreased cell growth and also played an essential role in the maintenance of the latency program in NPC cells.

### RNA-Sequencing of NOKs and PBMCs reveal strong transcriptional shifts of chemotactic and adhesion and a role for CXCR4 in EBV reactivation

**Fig. 7:**
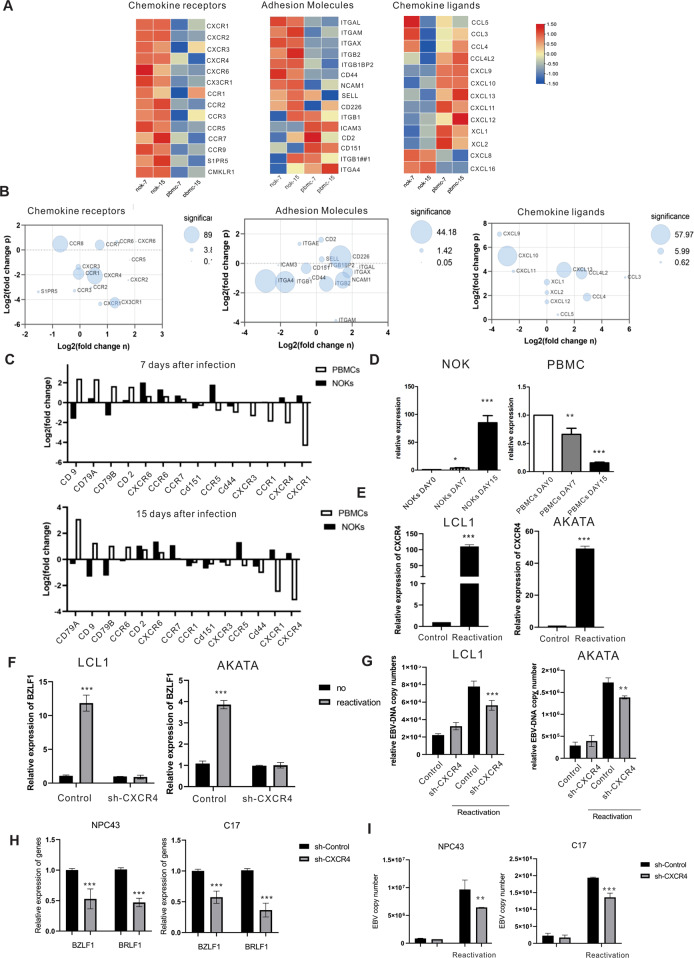
Differential gene expression of chemotactic and adhesion molecules. Differential gene expression of chemotactic and adhesion molecules showing that CXCR4 plays a critical role in virus reactivation. **A** Heat maps that report log2 values for the genes of chemokine related. **B** Bubble plots that display genes that are graphed **A**. **C** Surface expression phenotype on expanded EBV infected NOKs and PBMCs, compared to mock infected cells. **D** Validation of CXCR4 expression by qRT-PCR. **E** CXCR4 expression in reactivation of LCL1 and AKATA cells with qRT-PCR. The expression of CXCR4 increased from latent to reactivation phase. **F** Quantification of BZLF1 mRNA before and after the reactivation with TPA and Butyrate from three replicate experiments is shown. **G** EBV DNA copy number was detected by quantitative PCR in LCL1-shCXCR4 cell lines, LCL1-shControl, AKATA-shCXCR4 and AKATA-shControl cell groups before and after reactivation. **H** Quantification of EBV lytic gene BZLF1 and BRLF1 mRNA from three experiments is shown in NPC43-shCXCR4 cells and C17-shCXCR4 cells. **I** EBV DNA copy number was detected by qPCR in sh-CXCR4 and control groups before and after reactivation with TPA and Butyrate in NPC43 and C17 cells.

The cell surface molecules of the host cells are well known to be important for binding and fusion of the virus particle to cells and facilitate viral infection. From the GSEA analysis above, the results showed that the chemokine signaling pathway and cell adhesion molecules were strongly affected in both NOKs and PBMCs. Therefore, we further examined the differential gene expression of cellular chemotactic and adhesion genes. Our RNA-seq data showed that among the largest, and most statistically significant differences, we observed the downregulation of CXCR4 and CXCR1 in PBMCs. Specifically, CXCR4 was downregulated approximately 3-fold on PBMCs (Table 4). Remarkably, we also found a substantial increase in transcription of chemokine ligands XCL1, XCL2, CCL3, CCL4, and CCL5, and a significant loss in the transcription of CXCL8, CXCL16, and SIPR5 (Fig. 7A–C). Next, we validated the RNA-seq with RT-qPCR, and showed that CXCR4 expression was decreased in EBV infected PBMCs and significantly increased in EBV infected NOKs (Fig. 7D). We further monitored the expression of CXCR4 expression at the transcription level, and as expected the expression of CXCR4 was increased when LCL1 and AKATA cells were reactivated to induce their lytic cycle by 60 to 100 fold (Fig. 7E). Therefore, we asked if CXCR4 would play an important role in lytic reactivation. We knocked down CXCR4 and used TPA and sodium butyrate to reactivate LCL1 and AKATA cells. The lytic gene BZLF1 was expressed at a relatively high level in control cells when compared to shCXCR4 LCL1 and AKATA cell lines. Furthermore, a significant drop in the copy number of EBV genomes was seen when CXCR4 was knocked down (Fig. 7F, G). On the other hand, we also examined how CXCR4 may infect the lytic state of epithelial cells. We used lentivirus to knockdown CXCR4 in NPC43 and C17 cells and also confirmed the expression of CXCR4 with RT-PCR. The result showed that CXCR4 had no effect on NPC cell growth (Supplementary 4). However, CXCR4 is critical for expression of viral lytic gene transcription. BZLF1 and BRLF1 were significantly downregulated in the NPC-shCXCR4 cell lines compared to the Control. At the same time, the copy number of the EBV genome was also decreased in the NPC-shCXCR4 cell lines after the reactivation (Fig. 7H, I). These results strongly suggest that CXCR4 is a critical player in regulation of EBV reactivation from latency.

**Table 4:** Most significant differentially transcribed RNAs in PBMCs after 7 and 15 EBV infection.

	PBMCs		PBMCs	
	EBV (+) D7 VS EBV (−)		EBV (+) D15 VS EBV (−)	
Gene Name	Fold change	*P*-value	Fold change	*P*-value
**Upregulated**				
CCL22	4.50	5.69E-126	6.28	6.68E-216
RGS16	7.51	0	6.00	2.47E-191
CXCL10	5.36	1.84E-118	5.40	2.52E-82
MOXD1	4.58	3.75E-80	4.90	9.28E-68
CD80	4.26	1.11E-56	4.73	4.97E-55
LTA	4.86	2.63E-153	4.71	5.12E-94
CCL4L2	3.86	2.31E-51	4.70	6.15E-70
PCSK5	2.88	4.53E-151	4.60	1.35E-225
STRIP2	4.62	4.2E-132	4.56	2.34E-83
ZC3H12C	4.12	4.24E-113	4.53	5.48E-90
**Downregulated**				
MPEG1	−3.99	1.59E-06	−5.15	9.30E-10
IL7R	−3.75	7.85E-08	−4.39	2.34E-09
APLP2	−3.00	1.45E-06	−3.85	3.22E-08
Clorf162	−2.95	8.57E-09	−3.87	0.0012
TSC2203	−2.87	4.31E-05	−3.55	5.23E-13
Cllorf21	−2.53	3.45E-06	−3.39	2.24E-05
DMXL2	−2.48	9.84E-05	−3.29	1.71E-06
CXCR4	−2.22	2.73E-06	−3.14	7.20E-07
SGK1	−2.21	0.0001	−3.03	5.78E-07
TAGAP	−2.02	2.55E-06	−3.11	7.45E-05

## Discussion

**Fig. 8:**
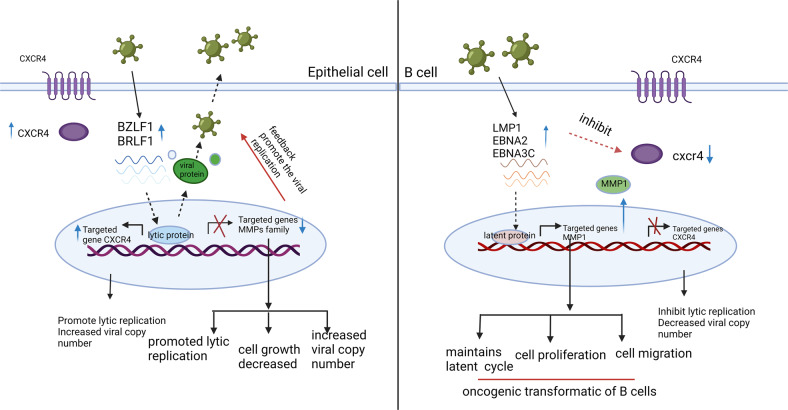
A schematic diagram shows that EBV can induce different expression patterns of MMP1 and CXCR4 and promotes different states of viral life cycle. A schematic illustrating the interaction between EBV infected NOKs and PBMCs. EBV transcripts were differentially expressed in NOKs and PBMCs, and in turn they would induce different cellular responses through dysregulation of different pathways. This activation leads to the expression of different downstream genes including MMP1 and CXCR4 ranked among the top 10 significantly affected in NOKs and PBMCs, respectively. During the primary infection, we found that MMP1 was increased in PBMCs, which can promote and sustain the latent cycle in EBV positive B cells. However, we also found that CXCR4 was inhibited by the latent viral protein of EBV in EBV positive B cells, which resulted in inhibition of the lytic state and viral replication. The two genes show opposite expression when epithelial cells are infected, so they played a different role in these two different cell lines. For the EBV positive NPC cells, we found that cell proliferation was decreased following MMP1 knockdown in EBV positive cell lines. Meanwhile, EBV lytic gene expression was significantly increased. During our investigation of EBV lytic genes in NPC cells, CXCR4 knockdown resulted in decreased EBV lytic gene expression. However, CXCR4 had a significant impact on cell proliferation in NPC43 and C17 cell lines.

This study simultaneously profiled the expression of EBV and cellular genes in PBMCs and NOKs after EBV infection utilizing high-throughput RNA sequencing. Our data showed a distinct pattern of EBV and cellular gene expression and identified multiple significant pathways in EBV-infected NOKs and PBMCs. These findings provide us with new insights into our understanding of the mechanisms linked to oncogenesis in which EBV infection contribute to cell transformation and immortalization. The interaction between EBV, PBMCs and NOKs suggests that there are differences in how these cell types respond to activation of cellular processes and pathways. These changes lead to expression of many different downstream genes. Most prominently, MMP1 and CXCR4 ranked in the top 10 genes that were changed in PBMCs and NOKs. During primary infection, we found that MMP1 was increased in PBMCs, which can promote and maintain the latent cycle. However, we also found that CXCR4 was inhibited during latent infection in PBMCs, and resulted in inhibition of the lytic state and viral replication (Fig. 8). These two genes showed opposite expression patterns when epithelial cells are infected with EBV suggesting that they play a different role in these two different cell types and shed new light on the interaction of EBV and the two cell types known to be infected.

We compared EBV-encoded latent and lytic gene expression in PBMCs and NOKs after EBV infection. Previously, the global transcriptome changes in primary human resting B lymphocytes during EBV transformation for different days were measured [31], and the gene expression changes in NOKs on EBV infection and differentiation also investigated [32]. However, our study revealed that the expression of EBV genes post infection had a tremendous impact on the cellular gene expression profiles in both PBMCs and NOKs. This was illustrated by the significant differences in gene expression between EBV negative and EBV-infected PBMCs and NOKs, which demonstrated that EBV can mediate global transcriptional reprogramming in these two cell types. The data showed that a total of 2265 genes were significantly changed in both PBMCs and NOKs after EBV infection. As anticipated, these genes showed the opposite trend in terms of transcription profiles on EBV infection, which drives epithelial and B cells towards a different response after primary infection. These results provide clues as to how different host cells can respond in a distinctly different manner to EBV infection. The top differentially transcribed genes, which were increased significantly after 7 and 15 dpi in PBMCs were CXCL10, CCL22, CD80 and CCL4L2 (Table 4). These genes are involved in the innate immune response, indicating that EBV activates the defensive mechanism by upregulating the immune response, as will be with other virus-infected cells, that promote the innate immune activation including influenza virus and Zika virus [33–35]. Interestingly, these genes were either downregulated or had negligible change during infection of NOKs, which suggests that EBV can elicit a strong innate immune response in PBMCs but not in NOKs. Further studies are required to elucidate the mechanism.

We then analyzed the differentially transcribed genes to identify the cell signaling pathways that are involved. The biological pathways downregulated early in NOKs include ribosome components that was seen at 7 days and continued to decrease at 15 days post-infection, therefore suggesting selective downregulation of host protein expression by the virus. At later time points the cell cycle genes were also downregulated, which is consistent with the suppression of proteins involved in cell cycle regulation. Additional, the most profound impact of EBV on the target cells was changed in epithelial/ECM remodeling pathways. Two main families of metallo-proteinases, including the MMP and ADAMTS family are specialized in degrading the ECM [36]. MMPs are comprised of varied functional proteases and are known to modulate barrier function via junctional protein cleavage [37, 38]. Here we showed that MMP1 was significantly upregulated in PBMCs in response to EBV but was downregulated in NOKs. As it is known that degradation of the main components of the ECM is primarily carried out by MMP-1 [39]. Thus, it is reasonable to propose that EBV activate MMP1 in order to demolish the matrix barrier of tissues facilitating further tumour migration and invasion. Accumulating evidence indicates that MMP1 may have additional functions, beyond its role in degrading collagen. We demonstrate that knock down of MMP1 induces expression of EBV lytic genes and downregulates the expression of EBV latent protein in LCL and EBV-positive AKATA cells, which indicated that MMP1 has an important role in maintaining a homeostatic equilibrium between B cells and the virus, being capable of keeping a strictly latent EBV cycle. Previous studies showed that viral proteins Zta and LMP1 can regulate the expression and activity of MMP-1 in NPC cells [40, 41]. In this study, we also provide mechanistic insights demonstrating that knock down MMP1 can induce EBV lytic gene expression at transcriptional level and also contributes to viral replication in NPC cells. In the meantime, we also observed that MMP1 promote the cell proliferation in late NPC cells. The discovery of different mechanisms involved in the regulation of MMP1 may reflect the complex pathway that ensure EBV latency in B cells and late NPC cells.

One of the most important host responses during viral infection is the production of antiviral cytokines and chemokines [42]. Similarly, EBV infection also induced changes in the chemokine pathway. Here we found that infection of EBV in PBMCs caused substantial downregulation of several chemokines, including CXCL16, CXCL8 and SIPR5 all of which play roles as mediators of the inflammatory response to many viruses. CXCL8 plays a role in attracting neutrophils to sites of inflammation and CXCL16 attracts CXCR6-expressing dendritic cells and T cells [43, 44]. These alterations could have an impact on multiple arms of the human immune system. It should be noted that a previous study showed lower CXCL8 gene expression in lymphoma lymph nodes when compared to reactive lymph nodes [45]. These findings provide a rationale to further investigate the full functional consequences of altered expression of CXCL8 and CXCL16 during primary infection and EBV reactivation. Moreover, our analysis has revealed that EBV induced upregulation of CCL4, CXCL9, CXCL10, CXCL11, CXCL12 and downregulation of CCR2, CCR3, CCR7, CXCR6, and CXCR4. However, this change showed an opposite trend in NOKs infection. Importantly, CXCR4 was one of the top 10 genes reduced in expression and showed the largest reduction. CXCR4 is an alpha-chemokine receptor specific for stromal-derived-factor-1 (SDF-1 also called CXCL12), a molecule endowed with potent chemotactic activity for lymphocytes [46, 47]. CXCR4 was shown to have high expression in many hematological malignancies [48–50], and multiple solid cancers [51, 52], which is also related to poor prognosis [50]. However, in our primary infection, CXCR4 expression was downregulated in infected PBMCs and upregulated in NOKs, albeit at a low level. Recent studies have shown that CXCR4 is regulated by many factors. Extracellular signalling (such as TGF-β) could increase CXCR4 expression in epithelial cells [19]. Nakayama et al. found that stable expression of EBNA2 and LMP1 downregulated CXCR4 expression in B cell lymphoma-derived cells [20]. BZLF1 expression controls the switch from latent to lytic infection. We knocked down CXCR4 with shRNA and detected an downregulation of BZLF1 and BRLF1 expression, suggesting that CXCR4 facilitating the early EBV lytic gene expression. Furthermore, inhibition of CXCR4 expression in B cells and NPC cells was beneficial to maintain EBV latency. CXCR4 could increase the EBV DNA copy number and induce more lytic EBV products BZLF1 and BRLF1A after the reactivation, which confirms the hypothesis that CXCR4 partly contributes to EBV reactivation. Subsequently, it was found that the inhibition of CXCR4 had no effect on cell proliferation in NPC cells (Fig. S1). CXCR4 may therefore be involved in the regulation of viral replication and may require the contribution of several viral proteins to dysregulate the host defense mechanism. However, additional experimental studies will explore these questions and provide a deeper understanding of the regulatory mechanism.

Thus, our genome-wide gene expression analysis by RNA-seq of PBMCs and NOKs offer an excellent model to reveal the similarities and differences in EBV infection of B cells and epithelial cells. This excellent experimental model used to explore more physiological microenvironment on EBV infection highlights some of the key signaling pathways affected by EBV infection, supports some previous findings, but has also identified new genes affected upon EBV infection. Further studies will provide new clues as to a more detailed molecular mechanism through which different host cells respond to EBV infection.

## Supplementary information

supplemental information

Reproducibility checklist

Original Data File

## Data Availability

All data generated or analyzed during this study are available from the corresponding author on reasonable request.
