# Supplementary material for: Transcriptome reprogramming of Epstein-Barr virus infected epithelial and B cells reveals distinct host-virus interaction profiles
Source: Cell Death Dis. 2022 Oct 22;13(10):894. doi: 10.1038/s41419-022-05327-1 (PMC9588026; doi:10.1038/s41419-022-05327-1)
Supplement: Supplementary file 1 — supplemental information [file 41419_2022_5327_MOESM1_ESM.docx]

**Supporting Information**

**S1 Figure. CXCR4 knock-down did not affect cell proliferation in NPC43 cells and C17 cells.**

(A) Cell proliferation fluorescence microscopy images of NPC43-shControl and NPC43-shCXCR4 cells after 5 days. (B) The expression of endogenous CXCR4 in NPC43-shControl and NPC43-shCXCR4 cells was determined by Q-pcr. (C) Cell growth curves of NPC43-shControl and NPC43-shCXCR4 cells determined by cell count after knockdown at different days. (D) Cell proliferation fluorescence microscopy images of C17-shControl and C17-shCXCR4 cells after 5 days. (E) The expression of endogenous CXCR4 in C17-shControl and C17-shCXCR4 cells was determined by Q-pcr. (F) Cell growth curves of C17-shControl and C17-shCXCR4 cells determined by cell count after CXCR4 knockdown at different days.

**S1 Table. Summary of primers used for Real-time PCR in this study.**

**S2 Table. EBV gene expression in PBMCs after EBV infection**

**S3 Table. EBV gene expression in NOKs after EBV infection**

**S1 Figure. CXCR4 knock-down did not affect cell proliferation in NPC43 cells and C17 cells.**


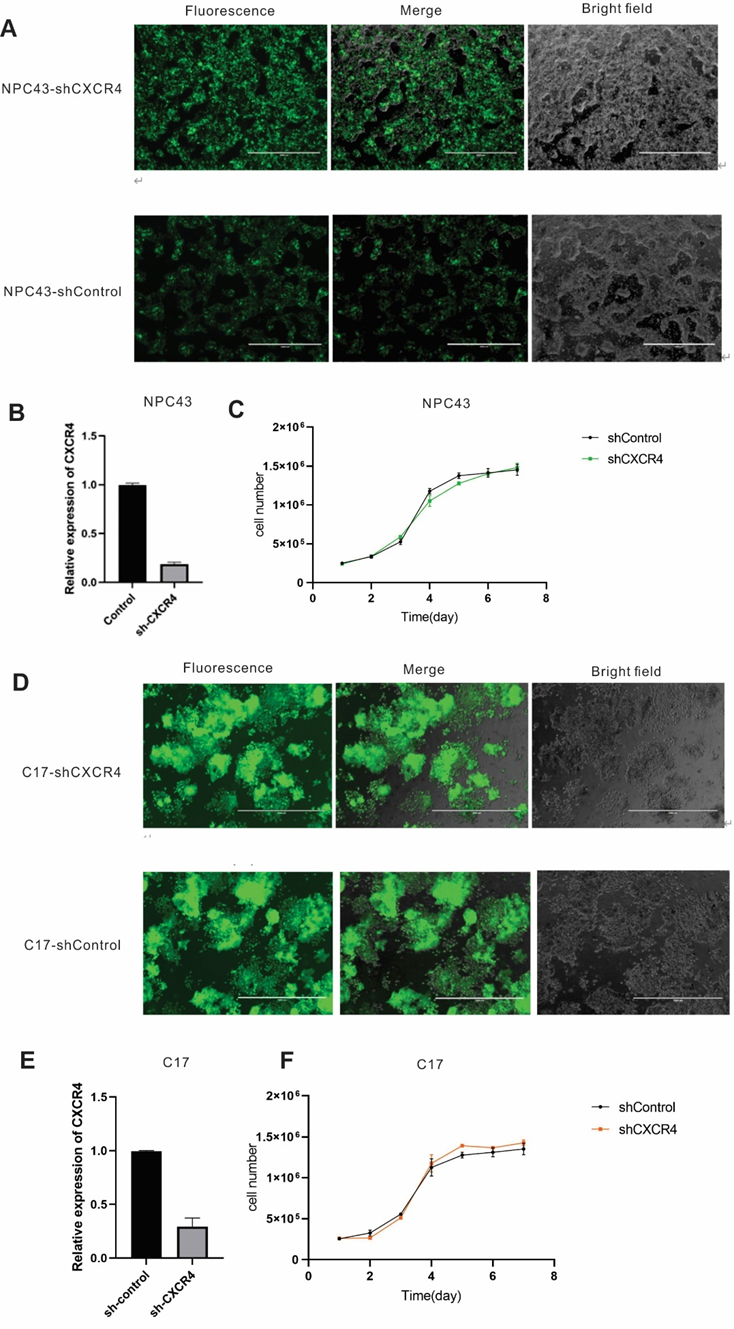


**Supplementary Table 1. Summary of primers used for RT-qPCR in this study.**

| **Gene** | **Forward sequence** | **Reverse sequence** | **Size, bp** |
| --- | --- | --- | --- |
| MMP1 | 5’- AGTGACTGGGAAACCAGATGCTGA -3’ | 5’- GCTCTTGGCAAATCTGGCGTGTAA -3’ | 162 |
| CXCR4 | 5’- GCCTTATCCTGCCTGGTATTGTC -3’ | 5’- GCGAAGAAAGCCAGGATGAGGAT -3’ | 130 |
| BZLF1 | 5’-CCAGGTTGAGGTGCTTCTCCCCCG -3’ | 5’-AACCGCTCCGACTGGGTCGTGGTT -3’ | 127 |
| EBNA2 | 5’-CGGGTTCTGGACTATCTGG -3’ | 5’- GAACTTCAACCCACACCATC-3’ | 104 |
| LMP1 | 5’- TAACTCCAACGAGGGCAGAC-3’ | 5’- GTGCGCCTAGGTTTTGAGAG-3’ | 90 |
| BRLF1 | 5’-AAGGCCTCCTAAGCTCCAAG -3’ | 5’-CCTGTCTTGGACGAGACCAT -3’ | 92 |
| EBNA3C | 5′-AGAAGGGGAGCGTGTGTTGT-3′ | 5′-GGCTCGTTTTTGACGTCGGC-3′ | 153 |
| GAPDH | 5′-TGCACCACCAACTGCTTAG-3′ | 5′-GATGCAGGGATGATGTTC-3′ | 176 |

**Supplementary Table 2. EBV gene expression in PBMCs after EBV infection**

| Gene name | Mean expression level (CPM) | | Gene name | Mean expression level (CPM) | |
| --- | --- | --- | --- | --- | --- |
|  | Day 7 | Day 15 |  | Day 7 | Day 15 |
| LMP-1 | 291272.07 | 276162.79 | BILF1 | 4863.06 | 16860.46 |
| BHRF1 | 104171.99 | 81395.34 | BFRF3 | 4869.41 | 2325.58 |
| EBNA-2 | 53493.72 | 53488.37 | BFRF2 | 4871.27 | 7558.13 |
| BHLF1 | 34553.36 | 19767.44 | BaRF1 | 4875.95 | 5822.01 |
| BcLF1 | 31737.90 | 23255.81 | BLRF1 | 4607.11 | 582.19 |
| EBNA-3C | 31481.95 | 26162.79 | BFLF2 | 4351.16 | 7568.89 |
| EBNA-3B | 24827.23 | 19771.41 | BFRF1 | 4364.48 | 4075.01 |
| BKRF4 | 23803.42 | 12790.69 | BVRF1 | 4343.71 | 4061.29 |
| EBNA-3A | 22011.77 | 14534.88 | BGLF3 | 4095.21 | 0 |
| BXLF2 | 17916.56 | 20930.23 | BALF5 | 4091.98 | 22674.41 |
| BORF2 | 17404.65 | 12209.30 | BRLF1 | 4099.37 | 3499.35 |
| BLLF1 | 16124.90 | 21511.62 | BBLF2/BBLF3 | 4102.08 | 5244.59 |
| BBRF3 | 14077.29 | 2906.97 | BBLF4 | 3583.31 | 6976.74 |
| BPLF1 | 13821.34 | 28488.37 | RPMS1 | 3592.53 | 2332.11 |
| BXLF1 | 13053.49 | 9302.32 | BORF1 | 3327.36 | 4069.76 |
| BKRF3 | 12029.69 | 8720.93 | BRRF1 | 3071.41 | 5232.55 |
| BcRF1 | 10238.03 | 9302.32 | BLLF3 | 2815.45 | 5876.22 |
| BILF2 | 9982.08 | 11046.51 | BSRF1 | 2829.14 | 2339.64 |
| BMRF1 | 9573.38 | 15116.27 | BGLF4 | 2303.55 | 3488.37 |
| BDLF3 | 9470.18 | 8720.93 | BKRF2 | 2347.60 | 1160.29 |
| BGLF2 | 9233.54 | 10465.11 | BGLF1 | 2028.29 | 2906.97 |
| BMRF2 | 9214.23 | 8139.53 | BDLF4 | 1791.65 | 3468.24 |
| BGRF1/BDRF1 | 9325.87 | 13372.09 | BBRF2 | 1842.14 | 2910.11 |
| oriLyt | 8446.37 | 5813.95 | BARF1 | 1734.39 | 1751.59 |
| EBNA-1 | 7912.47 | 11627.90 | BXRF1 | 1535.70 | 4651.16 |
| BOLF1 | 7856.98 | 7558.13 | BVLF1 | 1542.29 | 3459.36 |
| BSLF2/BMLF1 | 7835.22 | 8720.93 | LMP-2A | 1503.11 | 3514.35 |
| BLRF2 | 7678.52 | 7558.13 | BVRF2 | 1279.75 | 4649.25 |
| BNRF1 | 7422.5 | 12790.69 | BALF1 | 1023.80 | 1162.79 |
| BFLF1 | 7139.13 | 4088.54 | BBLF1 | 1049.81 | 573.55 |
| BZLF2 | 7166.62 | 4081.72 | Cp | 767.85 | 591.44 |
| BALF2 | 6928.77 | 9302.32 | BCRF1 | 511.90 | 6976.74 |
| BDLF1 | 6910.67 | 8720.93 | LF1 | 503.20 | 1744.18 |
| BGLF5 | 6695.03 | 4069.76 | BFRF1A | 525.58 | 1171.83 |
| BDLF2 | 6654.72 | 8139.53 | LMP-2B | 519.66 | 592.04 |
| BRRF2 | 5630.91 | 9302.32 | BGLF3.5 | 234.11 | 581.39 |
| BSLF1 | 5374.96 | 5822.19 | LF3 | 273.32 | 0 |
| BALF4 | 5119.01 | 18604.65 | TR | 265.15 | 0 |
| BBRF1 | 5123.92 | 2325.58 | oriP | 0 | 3482.17 |
| BTRF1 | 5128.62 | 5813.95 | BDLF3.5 | 0 | 1170.41 |
| BALF3 | 0 | 584.32 |  |  |  |

**Supplementary Table 3. EBV gene expression in NOKs after EBV infection**

| Gene name | Mean expression level (CPM) | | Gene name | Mean expression level (CPM) | |
| --- | --- | --- | --- | --- | --- |
|  | Day 7 | Day 15 |  | Day 7 | Day 15 |
| BPLF1 | 56831.45 | 53831.23 | BFRF1 | 9342.16 | 14548.98 |
| BKRF4 | 46126.90 | 43161.98 | BRLF1 | 9050.21 | 5819.59 |
| BcLF1 | 39704.17 | 44616.88 | BALF5 | 8952.90 | 8732.11 |
| BHLF1 | 39022.97 | 58680.89 | BBRF1 | 8855.59 | 11643.25 |
| BLLF1 | 36590.11 | 40737.15 | BORF1 | 7979.76 | 5819.59 |
| LMP-1 | 32600.23 | 42192.05 | BGLF1 | 7006.62 | 5826.79 |
| BDLF3 | 32502.92 | 27643.06 | BLRF1 | 6909.30 | 11651.37 |
| EBNA-3B | 26177.50 | 29582.93 | BMRF1 | 6520.05 | 6789.52 |
| BORF2 | 25301.67 | 26172.26 | BGLF4 | 6340.13 | 2909.80 |
| BILF2 | 24328.53 | 26188.17 | BGLF5 | 6325.42 | 5347.12 |
| EBNA-2 | 22382.25 | 24733.27 | BVRF1 | 6228.10 | 5334.63 |
| BGLF2 | 20533.28 | 17951.31 | BRRF1 | 5838.85 | 4364.69 |
| BDLF2 | 20144.02 | 17943.74 | BSRF1 | 5741.53 | 3879.73 |
| EBNA-1 | 19657.45 | 21338.51 | BGLF3 | 5060.33 | 1454.90 |
| EBNA-3C | 19462.83 | 15033.95 | BVRF2 | 4865.71 | 1939.86 |
| BKRF3 | 19170.88 | 19883.61 | BKRF2 | 4671.08 | 4849.66 |
| BOLF1 | 18878.94 | 20372.19 | LMP-2B | 4379.14 | 3394.76 |
| BNRF1 | 18587.00 | 20368.57 | oriP | 3989.88 | 8715.44 |
| BXLF2 | 17613.86 | 11639.19 | BaRF1 | 3995.13 | 5337.25 |
| EBNA-3A | 17516.54 | 12124.15 | BILF1 | 3795.25 | 1945.11 |
| BBRF3 | 17419.23 | 12609.12 | BVLF1 | 3790.14 | 4377.52 |
| BRRF2 | 16932.66 | 13094.08 | oriLyt | 3697.94 | 9212.08 |
| BGRF1/BDRF1 | 16835.34 | 14064.02 | BLLF3 | 3503.31 | 9699.32 |
| BLRF2 | 16640.72 | 15041.22 | BARF1 | 3211.37 | 969.93 |
| BALF4 | 15083.69 | 10184.29 | BBRF2 | 3206.11 | 4851.33 |
| BXLF1 | 14889.06 | 9214.35 | LMP-2A | 3119.29 | 1940.19 |
| BFRF2 | 14499.81 | 8729.39 | BXRF1 | 3114.05 | 2424.83 |
| BHRF1 | 14305.18 | 16973.81 | BDLF4 | 2919.42 | 2902.51 |
| BALF2 | 14110.55 | 14071.56 | BFRF1A | 2627.48 | 3390.08 |
| BFLF1 | 13526.66 | 15518.91 | BBLF1 | 2140.91 | 1931.20 |
| BDLF1 | 13234.72 | 16003.88 | BGLF3.5 | 2043.60 | 484.97 |
| BFLF2 | 12650.84 | 11639.19 | BCRF1 | 2032.72 | 971.08 |
| BcRF1 | 12367.39 | 9222.17 | Cp | 1946.28 | 2912.47 |
| BSLF1 | 12358.89 | 11154.22 | LF1 | 1751.65 | 2931.09 |
| BBLF4 | 11191.12 | 10669.25 | BALF1 | 1459.71 | 481.35 |
| BTRF1 | 10996.50 | 7274.49 | RPMS1 | 1362.40 | 961.57 |
| BFRF3 | 10217.98 | 12124.15 | BDLF3.5 | 973.14 | 960.04 |
| BBLF2/BBLF3 | 10229.67 | 8244.42 | BALF3 | 389.26 | 975.58 |
| BZLF2 | 10120.67 | 11639.19 | TR | 194.63 | 963.22 |
| BMRF2 | 10023.36 | 11645.22 | LF3 | 0.00 | 487.29 |
