# Supplementary figures and images for: Transcriptome reprogramming of Epstein-Barr virus infected epithelial and B cells reveals distinct host-virus interaction profiles
Source: Cell Death Dis. 2022 Oct 22;13(10):894. doi: 10.1038/s41419-022-05327-1 (PMC9588026; doi:10.1038/s41419-022-05327-1)

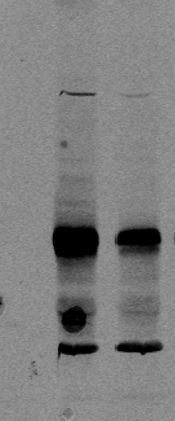

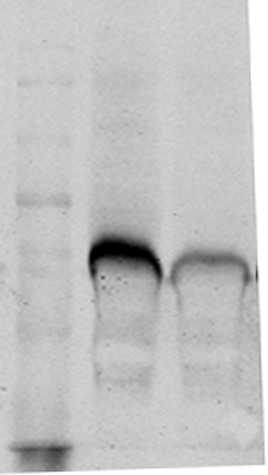

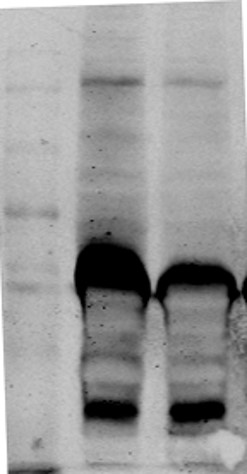

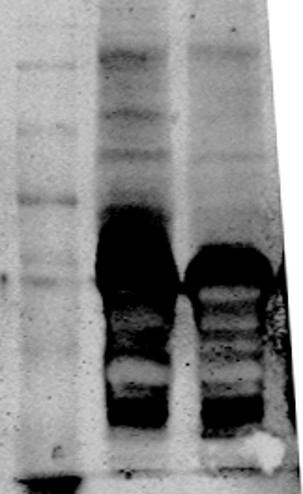

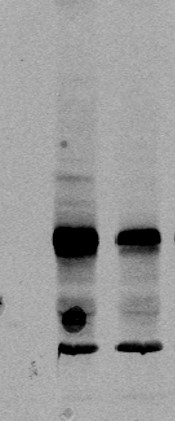

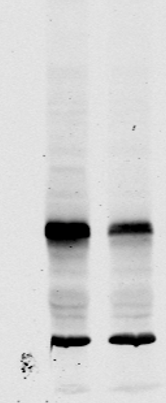

Supplement: Supplementary file 3 — Original Data File [file 41419_2022_5327_MOESM3_ESM.docx]
